# Nuclear morphologies: their diversity and functional relevance

**DOI:** 10.1007/s00412-016-0614-5

**Published:** 2016-09-08

**Authors:** Benjamin M. Skinner, Emma E. P. Johnson

**Affiliations:** Department of Pathology, University of Cambridge, Cambridge, CB2 1QP UK

**Keywords:** Shape, Gene expression, Eukaryote, Differentiation, Chromatin

## Abstract

Studies of chromosome and genome biology often focus on condensed chromatin in the form of chromosomes and neglect the non-dividing cells. Even when interphase nuclei are considered, they are often then treated as interchangeable round objects. However, different cell types can have very different nuclear shapes, and these shapes have impacts on cellular function; indeed, many pathologies are linked with alterations to nuclear shape. In this review, we describe some of the nuclear morphologies beyond the spherical and ovoid. Many of the leukocytes of the immune system have lobed nuclei, which aid their flexibility and migration; smooth muscle cells have a spindle shaped nucleus, which must deform during muscle contractions; spermatozoa have highly condensed nuclei which adopt varied shapes, potentially associated with swimming efficiency. Nuclei are not passive passengers within the cell. There are clear effects of nuclear shape on the transcriptional activity of the cell. Recent work has shown that regulation of gene expression can be influenced by nuclear morphology, and that cells can drastically remodel their chromatin during differentiation. The link between the nucleoskeleton and the cytoskeleton at the nuclear envelope provides a mechanism for transmission of mechanical forces into the nucleus, directly affecting chromatin compaction and organisation.

## Introduction

A nucleus is not just a ‘bag of holding’ for chromatin. It is a complex and dynamic organelle within a eukaryotic cell, subject to layers of regulation and imposing its own effects onto the cell it lies within and the genes that lie within it. Yet the first image of a nucleus that many of us encounter in textbooks at school or university is of a spherical or ovoid object, holding DNA, quickly put to the side in favour of metaphase chromosomes. In reality, for many types of cells, it is true that the nucleus is spherical or ovoid. Fibroblasts, macrophages, lymphocytes, splenocytes, these all have that pattern, and are easy cell types to harvest for microscopy. No wonder, then, that many biologists do not get to see the variety.

Despite a considerable interest in nuclear shape and chromatin organisation over decades, and a wealth of new technologies for 3D visualisation, live cell imaging, sequencing-based structural imaging, we are just beginning to appreciate the connection between nuclear structure and function. We are now seeing how nuclear structure can be changed by the cell’s activity and environment, but we are also seeing that the morphology of the nucleus itself can impact gene expression. It is also comparatively recently that we have started to understand how chromatin is organised and distributed within these varied cell types—whether chromosomes occupy preferred locations or territories within the nucleus, as has been seen in spherical and ovoid nuclei for many years (Cremer and Cremer 2010).

In this review, we aim to outline some of the variations in nuclear shapes seen in different cell types (predominantly human or mammalian, but not entirely ignoring the rest of the eukaryotic world). We show how these shapes can play a functional role in the cell and give an overview of the link between nuclear morphology and transcriptional regulation.

## Examples of nuclear morphologies

**Fig. 1:**
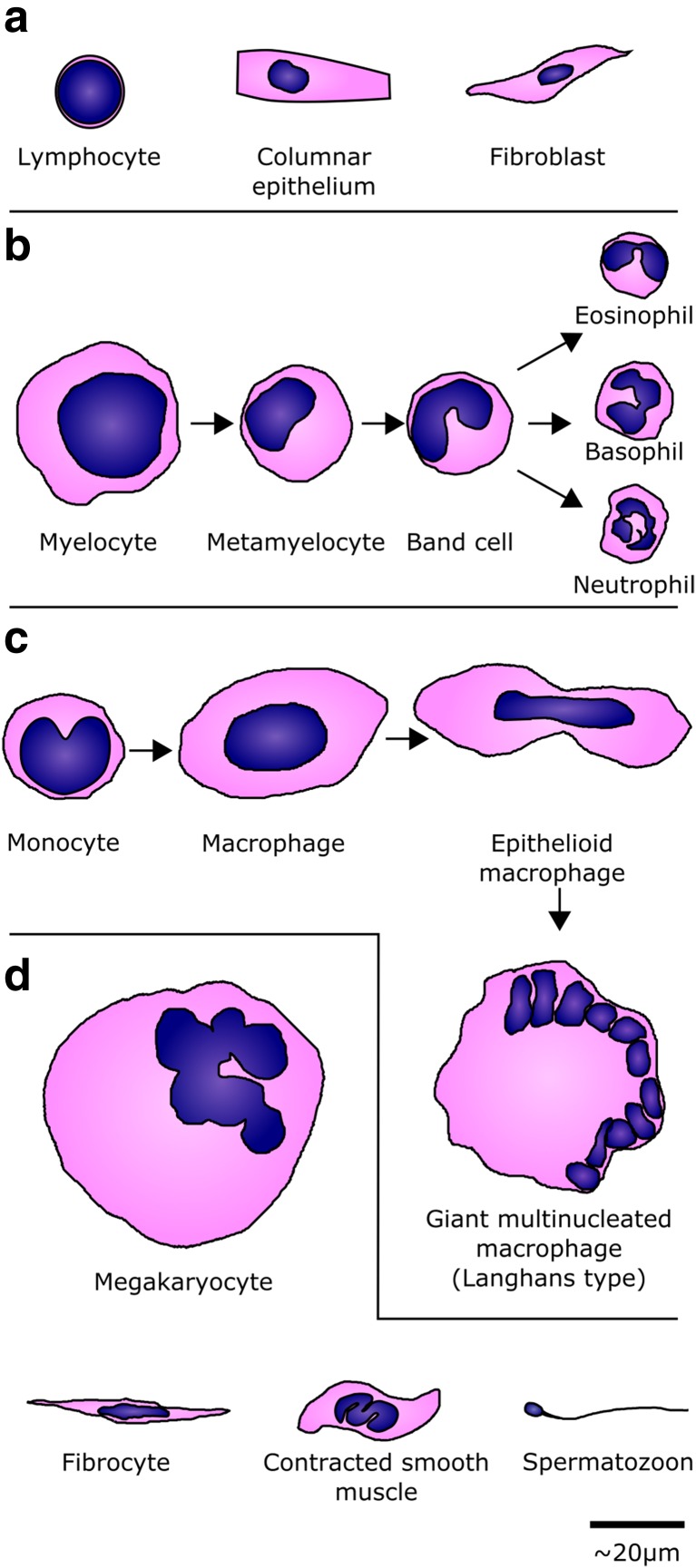
Examples of some of the human cell types mentioned in the main text. Nuclei are drawn in *blue* against the cytoplasm in *pink*. **a** Spherical and ovoid nuclei. **b** The lobed granulocyte lineage. **c** The lobed monocyte, and some of its differentiated macrophage stages. **d** Other shapes, including the polyploid megakaryocyte, fusiform fibrocyte and smooth muscle nuclei, and the condensed nucleus of a sperm

We provide here some selected examples of different nuclear morphologies and indicate what the functional relevance may be. Schematic drawings of example cells are given in Fig. 1, drawn approximately to scale. Most of the examples shown in the figure are from humans; readers should bear in mind that cell types and shapes may also differ between species.

Despite the focus here on non-spherical nuclei, the nuclei of cell types that are predominantly spherical or ovoid are not homogeneous internally; there are many different patterns by which the chromatin within the nucleus may be organised, which relates to the functional status of the cell. Nuclear size also appears to be strongly influenced by the size of the cell itself, via the ratio of nuclear volume to cytoplasmic volume (Huber and Gerace 2007). This correlation between nuclear size and cell size is thought to be able to drive genome size reduction in particular lineages—for example birds and bats, which require highly efficient metabolisms for the energy requirements of flight (Smith and Gregory 2009). Nonetheless, we still see variation in nuclear size in terminally differentiated cells depending on the condensation and organisation of the chromatin and the activity of the cell, from large polyploid hepatocytes to tiny spermatids.

## Lobed or segmented nuclei

### Granulocytes of the immune system

Vertebrate immune systems contain a variety of white cells from the myeloid lineage, termed granulocytes for their cytoplasmic appearance under haematoxylin and eosin dye. The granulocytes have been commonly recognised and distinguished histologically by their nuclear shapes and sizes. They contain multi-lobed nuclei, each lobe connected by a short region of nucleoplasm (Fig. 1b). Of the granulocytes, eosinophils have the fewest lobes. Their bi-lobed nucleus together with their intense eosin staining means they are often described to histology students as a sunburned face wearing dark sunglasses. Much of the variation within each cell type is found in the number of lobes; an increased lobe number is termed hypersegmentation. Hypersegmentation of eosinophils is rare, but has been seen in acute eosinophilic pneumonia, with lobe number increased to three or four lobes (Maeno et al. 2000), and could be linked to stimulation with lymphokines (Chihara and Nakajima 1989). In basophils, hypersegmentation is also rare but has been occasionally observed (Xu 2014). However, most of the studies of granulocyte nuclear structure have been performed on the neutrophils.

### Neutrophils

Mammalian neutrophils—and avian or reptilian heterophils (Claver and Quaglia 2009)—have segmented, multi-lobed nuclei, usually containing between two and five lobes, separated by thin filaments of nucleoplasm with little to no internal chromatin. The lobed structure develops from a spherical myelocyte precursor, gradually increasing the number and prominence of lobes through the concave metamyelocyte and band cell stages to the mature neutrophil (Fig. 1b).

Chromosome painting and 3D analysis have shown that most chromosomes are randomly distributed within neutrophil lobes, but the organisation can change upon bacterial stimulation (Yerle-Bouissou et al. 2009; Mompart et al. 2013). Within each lobe, the chromatin organisation follows a general gene-density based arrangement, in which the gene-poor chromatin is located towards the nuclear periphery, and gene-dense chromatin more internal (Hübner et al. 2015). Curiously, the inactive X chromosome in women is frequently found in a terminal lobe, often with a distinct ‘drumstick’ appearance (Karni et al. 2001), and it appears that the position of the inactive X within the precursor myelocyte may determine the polarity of the neutrophil. It remains unknown how polarity is determined in XY neutrophils.

Hypersegmentation of neutrophils, to six or more lobes, is associated with megaloblastic anaemias, such as result from deficiencies in Vitamin B12 and folic acid, and iron deficiency anaemia (Westerman et al. 1999). It is also associated with Boucher-Neuhäuser syndrome (Umehara et al. 2010; Koh et al. 2015), a lipid metabolic defect. In rats, vitamin A deficiency caused hypersegmentation, linked to a requirement of retinoids for differentiation of promyelocytes to mature neutrophils (Twining et al. 1996). Consequently, there are clearly many pathways that contribute to the establishment of a lobed nuclear morphology. What though is it for?

### Functional significance of a lobed nucleus

It is thought that the lobular arrangement makes the nucleus easier to deform and, hence, help the neutrophils pass through small gaps in the endothelium and extracellular matrix more easily (Hoffmann et al. 2007); granulocytes with defects in lamin B receptors (a component of the inner nuclear membrane) are unable to adopt a normal segmented shape, have fewer lobes (Hoffmann et al. 2002), and are poorer at passing through these small spaces. Neutrophils also have a higher variability in the length of the linker DNA between nucleosomes than T-lymphocyte populations (Valouev et al. 2011), pointing to increased chromatin flexibility.

However, neutrophils are not the only migratory cell in circulation; circulating monocytes, for example, have a lobed nucleus but, as described below, the lobes are larger and fewer. Monocytes are also flexible enough to enter tissues, whereupon they differentiate into various other cell types including macrophages. Indeed, comparisons of the migration of monocytes and neutrophils suggest that the monocytes are at least equally flexible when penetrating basement membranes, and that neutrophil migration is aided by reorganisation of the extracellular matrix via proteolytic cleavage of laminins (Voisin et al. 2009). The circulating fibrocytes and lymphocytes mentioned below are also migratory and have spindle-shaped and spherical nuclei, respectively.

Consequently, while the lobular shape of neutrophils may aid migration, is not strictly necessary for migration. Why then should neutrophils adopt lobes, when other cells do not? Perhaps the answer lies in the lifespan of the cells. The half life of a neutrophil in circulation is about 6 h (Summers et al. 2010). Though circulating monocytes live only a couple of days, macrophages may live for months in a tissue, as can lymphocytes.

The granulocytes have lower lamin protein content than macrophages or monocytes—predominantly a loss of the lamins A and C, with an increase in lamin B (Hoffmann et al. 2007). The lamin proteins, as described in more detail later, provide structural support to the nucleus, and protect against damage from mechanical stresses. Particularly, the ratio of lamin A:B balances the stiffness of the nucleus against its elasticity (Shin et al. 2013). Correspondingly, defects in the lamins associated with normal aging affects nuclear shape in all the granulocytes (Scaffidi et al. 2005; Chan et al. 2010), a result of changes to the stiffness and structure of the nuclear lamina. These age-related structural defects are also seen in laminopathies such as Hutchinson-Gilford Progeria Syndrome (Worman and Courvalin 2005).

Furthermore, rats treated with cyclophosphamide (a DNA cross-linker that disrupts DNA replication) have hypersegmented tetraploid neutrophils in their blood (Kotelnikov et al. 1988). The underlying mechanism driving hypersegmentation seems to be both failures during DNA synthesis and DNA damage or loss of nuclear structural integrity. Consequently, it appears that the extra flexibility of neutrophil nuclei comes at the cost of lowering their lifespan (Harada et al. 2014), a cost that other, longer-lived cell types cannot bear.

### Neutrophil extracellular traps

Neutrophils are capable of a form of cell death termed ‘NETosis’. They produce meshes of chromatin complexed with cytoplasmic proteins, termed Neutrophil Extracellular Traps (NETs), which capture bacteria (Brinkmann and Zychlinsky 2007; Brinkmann and Zychlinsky 2012). Such traps have been seen in orthologous cell types across vertebrates. During the process of NET formation, the nucleus loses its lobular structure, and the chromatin decondenses. The nuclear and cell membranes break down, releasing the NET into the extracellular space over ∼1–4 h. In particular circumstances, such as in response to *Staphylococcus aureus*, neutrophils may be able to generate NETs without lysis of the cell, by generating chromatin-filled vesicles that rupture after budding, a process that can happen in only minutes to an hour (Pilsczek et al. 2010).

Interestingly, NETs (or equivalents) can be produced by other leukocytes in addition to neutrophils (Goldmann and Medina 2013), such as mast cells and eosinophils. It remains unclear whether the lobular structure of the granulocyte nucleus is relevant for the formation of NETs (Veda 2011), and studies are needed to test the effects of the reduced structural stability of the nucleus on the speed or ease with which NETs can be formed.

### Monocytes and macrophages

Monocytes have a bilobed nucleus (Fig. 1c), which frequently presents in tissue sections and blood smears as a U- or kidney-shaped nucleus. The lobed structure arises in promonocytes, where an initial spherical nucleus acquires an indentation that develops into the separation of the lobes (Fawcett 1970). The reason for the lobed structure is still unclear; perhaps it helps with the flexibility of the nucleus, but leaves the nucleus less susceptible to damage than the highly segmented granulocytes.

The nucleus generally becomes more rounded following recruitment into tissues and further differentiation into a variety of macrophages and other cell types (Mosser and Edwards 2008). At high resolution, a clear difference is observable in the chromatin distribution within the nuclei. Chromatin domains within monocytes are aggregated into clusters, with channels and spaces between them. In monocytes—and indeed granulocytes—the channels and spaces within the nucleus are large, and may facilitate chromatin deformation upon migration (Hübner et al. 2015).

Even after differentiation into a macrophage, the cell nucleus can undergo extensive deformation in response to environmental conditions. Examples of nuclear reshaping of macrophages can be seen in electron microscopy images (Sato-Nishiwaki et al. 2013), and the nucleus is both displaced with the cell and reshaped from round to kidney-shaped in response to *Bacillus anthricis* edema toxin (Trescos et al. 2015). It is worth noting that macrophages remain functionally plastic—they can change between roles with relative ease (Mosser and Edwards 2008), and perhaps the readily deformable nucleus facilitates this via impacts on transcriptional regulation.

Many questions remain about these cells. Individual macrophages can fuse into giant macrophages (see Fig. 1c), thought to improve the efficiency of phagocytosis (McNally and Anderson 2011). Electron microscopy images show dense packing and distortion of abutting nuclei in giant cells (Sutton and Weiss 1966), but how do these shapes affect function and what is the relevance of nuclear position within these cells, such as the Langhans-type giant cells in which nuclei form a horseshoe around the periphery?

### Megakaryocytes

Megakaryocytes are the precursor cells from which platelets will develop by fragmentation of the cytoplasm. Their large multilobed nuclei are produced by successive rounds of endomitosis—that is, cell division in which the mitotic cycle stops during anaphase, skipping telophase and cytokinesis (Patel et al. 2005). This results in a large nucleus with a variable DNA content from 4 to 128 N.

In contrast to granulocytes, the lobes appear clustered, like a bunch of grapes, rather than separated by strands. Furthermore, there appears to be a difference in chromosomal segregation patterns between high and low ploidy cells (Papadantonakis et al. 2008). Although the nuclei are variable in morphology between cells, there are some clear morphological appearances that can be used to identify pathologies. For example, chronic myeloproliferative disorders are often accompanied by irregularities in morphology, and increased variation in lobe number (Ballarò et al. 2008), probably a symptom of disruptions to the structure of the nuclear envelope. Multinucleated megakaryocytes, as can arise in dysplasias, appear to arise from a further progression through the mitotic cycle (Münch et al. 2011).

It remains uncertain what the functional relevance of the ploidy or lobulation is in megakaryocytes; they exhibit functional gene expression amplification resulting from the polyploidy, but studies attempting to link platelet formation with ploidy and morphology have yielded inconclusive results to date (Machlus and Italiano 2013). Another common mammalian polyploid nucleus, that of the hepatocyte, is not lobed, but tends only to reach 8 N. Consequently, it remains unclear whether the lobulation is a physical response to the greater ploidy, or a result of inherited differentiation or programming pathways shared with the granulocyte lineages.

## Fusiform (spindle-shaped) nuclei

Before describing some cells with spindle shaped nuclei, we must mention the importance of the local environment of the cell in establishing and controlling the shape of the nucleus; many of the nuclei seen adopting fusiform shapes may also be found with a more spherical morphology dependent on tissue state and cell density, as we discuss in the later section on control of nuclear shape.

### Fibrocytes

Fibrocytes are hematopoietic lineage cells derived from peripheral blood mononuclear cells, or from CD14-positive monocytes (Curnow et al. 2010). They are associated with the inflammatory response and are actively recruited to sites of wounds (Metz 2003; Suga et al. 2014). Fibrocytes are capable of expressing α-smooth muscle actin (Quan et al. 2004) and extracellular matrix, and migrate into tissues prior to differentiation into (among other cell types) myofibroblasts. Subsequent to differentiation, they become almost indistinguishable to fibroblasts, and thus, imaging these cells has proved challenging (Suga et al. 2014).

Fibrocytes have spindle shaped cells and nuclei, which in the absence of differentiation appear stable in culture (Hong et al. 2005). As a migratory cell type, their spindle-shaped nucleus is intriguing, yet to date little is known about the biomechanics of their nuclei, their stiffness, or indeed whether the spindle shape is maintained during migration.

### Mesenchyme: smooth muscle

A further example of fusiform nuclei may be seen in smooth muscle. These cells have spindle-shaped nuclei embedded within the muscle fibre (contrasting with skeletal muscle, where the nuclei lie outside the fibre). This means that the nuclei are themselves subject to contraction, and the spindles are squeezed into a corkscrew shape as the muscle contracts—see, for example, lovely images of isolated contracted nuclei from Franke and Schinko (1969). Nagayama et al. (2011) demonstrated actin stress fibres attached to the outside of the nucleus, which are thought to mediate the contraction of the nucleus, stabilise the shape during relaxation, and to control the position of the nucleus within the cell.

### Endosperm in flowering plants

Fusiform nuclei are seen in the syncytial endosperm of many flowering plants such as *Arabidopsis thaliana* and *Coronopus didymus*, in which the nuclei are surrounded by a cage of microtubules. As the endosperm develops, and begins to cellularize, an actin skeleton associates with the nucleus in addition to the microtubules. Eventually, the nuclei adopt a more spherical shape, again apparently mediated by the surrounding microtubule network (Nguyen et al. 2001). At this later stage, the chromatin organisation within the nucleus appears to favour pairwise associations between chromosomes which may facilitate epigenetic regulation (Baroux et al. 2016). However, the relationship between the morphological changes and the functional organisation of the nucleus is poorly understood at this point.

## Spermatozoa

Perhaps one of the best studied cell types with an asymmetric nuclear shape is spermatozoa. There is a dramatic distinction between male and female gametes across metazoa; while the ovum is (usually) large, immotile and has a spherical nucleus (e.g., Zuccotti et al. 2005), the spermatozoa are small, highly motile and have an array of shapes. The iconic tadpole-shape is only one of many solutions evolution has crafted in the task of making cells that can swim energetically and carry a streamlined payload of DNA.

During the process of spermiogenesis, histones are replaced with protamines, enabling a greater compaction of the chromatin. The reasons for this extra compaction are debated; it likely aids swimming ability, but may also help protect the DNA from damage, and provide an extra level of epigenetic regulation to the paternal genome (Rathke et al. 2014).

As the nucleus compacts, the developing spermatid also sheds most of its cytoplasm. Consequently, the majority of the head is filled by the sperm nucleus, and the shape of the nucleus often closely follows the shape of the sperm head. It seems that the nucleus is an active participant in the development of the final sperm shape—the nucleus condenses and adopts a shape before the cytoplasm is lost and the cell membrane tightens in, rather than the condensing cell squeezing the nucleus into shape; see for example the staged spermatids in Russell et al. (1993).

Although all sperm require the ability to swim, it appears there is no single most efficient shape for this. The swimming efficiencies of a given shape also depend on environmental factors, such as the medium through which the sperm will be travelling; conditions are quite different for the sperm of sea urchins released into the ocean, to opossum sperm swimming through a viscous fluid and requiring a double-headed sperm to maintain orientation (Moore and Taggart 1995).

### Examples of distinctive sperm shapes

**Fig. 2:**
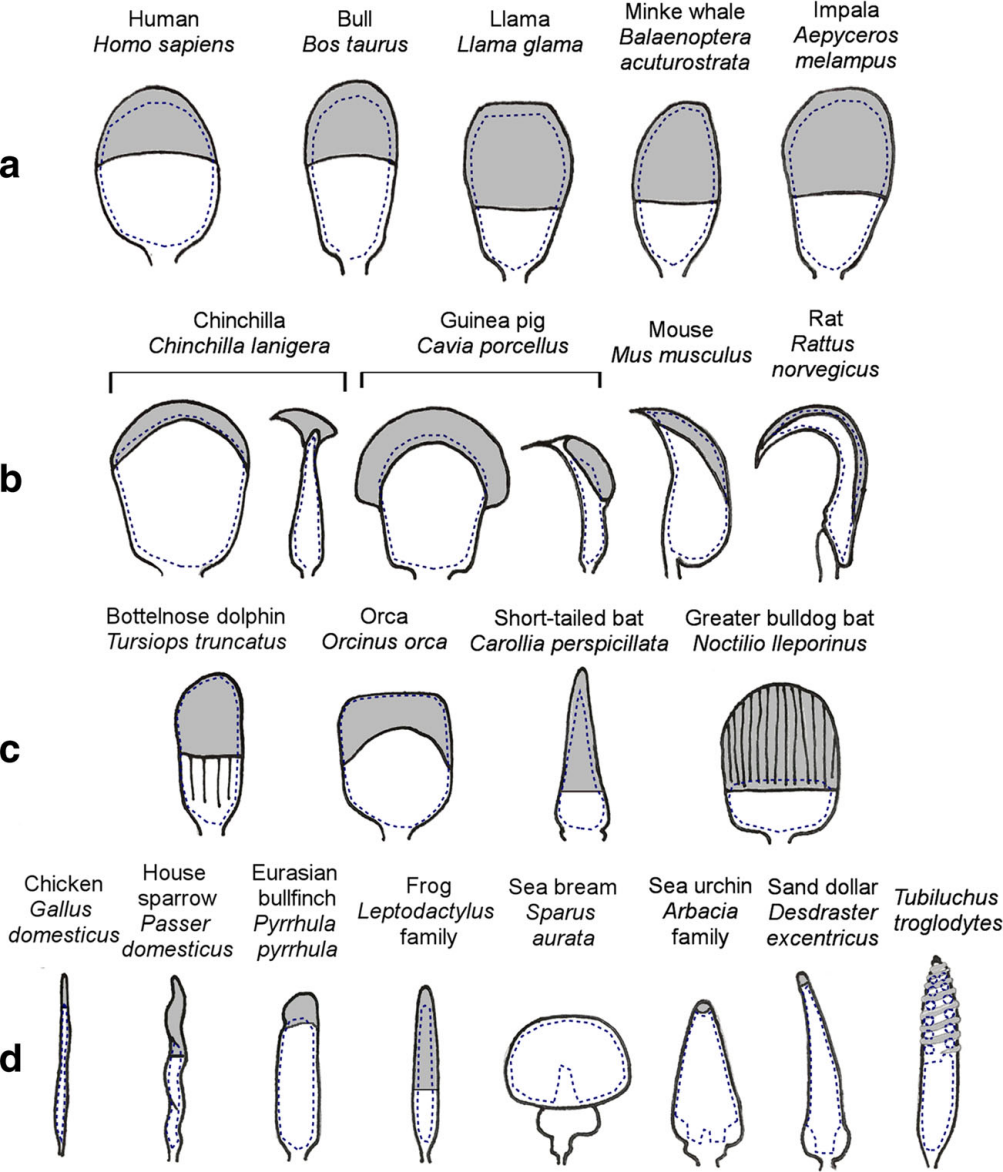
A selection of sperm head morphologies from across metazoa; acrosomal regions are shaded in *grey* and nucleus cross-sections denoted by a *dashed outline*. **a** The typical ovate or paddle head shape seen in many mammals. **b** Examples of giant acrosomes (including sagittal cross-sections) and falciform hooks seen in rodents. **c** Atypical mammalian head shapes. **d** Examples of morphologies from outside mammalia, including the anomalous sperm head of the Eurasian bullfinch (*Pyrrulah pyrrulah*), the rounded acrosome-less sperm head of the sea bream (*Sparus aurata*) and the spiralling acrosome sperm head of the ‘living fossil’, *Tubiluchus troglodytes*—the nucleus of which also forms a remarkable double spiral in the anterior portion of the sperm head, seen here in cross-section

Mammalian spermatozoa commonly conform to the stereotypical ‘tadpole’ or ‘paddle’ shape. They possess a head partly covered by an enzyme containing region (the acrosome), a neck, midpiece, a tail of some length, and are dorsoventrally flattened to a degree. However, even within mammals, an assortment of sperm shapes, especially relating to the head, can be observed (examples of sperm head shapes across a variety of taxa are given in Fig. 2); from the ovate-like shape of pig and human sperm, to the falciform sperm head of rodents, the ensiform sperm heads seen in some species of bats (Beguelini et al. 2014), and the more square-headed sperm of orcas and beluga whales (Miller et al. 2002).

Sperm heads also vary in the relative shapes and sizes of their functional regions; the acrosome is a region located over part of the anterior half of the head, which contains enzymes necessary to engage, disperse and penetrate the strata surrounding the ovum. A number of mammalian species, including the guinea pig (*Cavia porcellus*) and ground squirrel *(Otospermophilus beecheyi*) (Fawcett 1970), various species of shrew (Bedford et al. 1994), and the greater bulldog bat (*Noctilio leporinus*) (Phillips et al. 1997) have been observed to produce sperm with giant, and often curiously shaped, acrosomes. Despite the variation in size and shape, generally, there is a correlation between sperm size, number and fecundity (Gomendio and Roldan 2008).

### Sperm shape variation between taxonomic groups

A wealth of scanning electron microscope images of sperm were produced between the 1960s and 1990s. This technique allows for detailed examination of sperm ultrastructure including substructures of the sperm head, such as the nucleus. However, studies comparing sperm shape between species and other taxonomic groups seem surprisingly rare, with work often focused on the detailed examination of the sperm of a single species.

Some studies reveal remarkable examples of outliers in spermatozoan architecture: within passerine birds, the Eurasian bullfinch (*Pyrrhula pyrrhula*) is identified as an oddity (Birkhead et al. 2007) due to the chunkier tubular shape of its sperm when compared to the typical passerine worm-like, spiralling sperm head shape (see Fig. 2). The greater bulldog bat (*Noctilio leporinus*) possesses sperm described as ‘unique among mammalian spermatozoa’, owing to their spatulate, ridged and giant sperm acrosome. Unusually, the condensed nucleus only occupies roughly one third of the sperm head (Phillips et al. 1997). The variation of sperm shapes is additionally extraordinary when looking beyond the vertebrates. One remarkable example is the double-helical nucleus of the psudocoelomate worm *Tubiluchus troglodytes*, around which the acrosome also spirals (Ferraguti and Garbelli 2006).

The origins of sperm head shapes are not always well understood, and with such variation seemingly being generated over a relatively short evolutionary time period, sperm morphology may provide additional clues in the search for the origins and relatedness of even minor taxonomic groups (Rowe et al. 2015). Although uncommon, such detailed and digestible comparisons of sperm shape across taxa include the work of Downing Meisner et al. (2005), who examined sperm from 36 species of aridactylans, perissodactylans and cetaceans and outlined the somewhat subtle variation within the broadly elongate ovate sperm morph of these groups. Other comparisons include fish (Jamieson and Leung 1991), Asian rodents (Breed and Yong 1986; Breed and Musser 1991), and passerine birds (Birkhead et al. 2006); but whilst these works focus primarily on the potential of sperm shape for use in attaining phylogenetic clarity, it is often beyond their scope to do anything more than postulate the origin and functional relevance of nuclear shape variations within these groups, or to consider the processes by which these shapes arise.

### Influences on sperm shape

Mating and post-copulatory preferences exert pressure on sperm. Within promiscuous species and species with sperm-storing females, sperm may compete directly with rival male sperm as well as sperm from the same ejaculate. In the eusocial naked mole rat, reproduction is restricted to one dominant female queen and a single breeding male, suggesting limited or no sperm competition between males. Extreme sperm polymorphism is seen within a single ejaculate, including lobed, compressed, double-headed, and miniaturised sperm heads. Sperm exhibit poor motility, sperm concentration is highly varied between males, and defining a ‘normal’ spermatozoa morphology is difficult (van der Horst et al. 2011); this examination of sperm from across the colony structure also suggested that the sperm had irregular and variable chromatin condensation.

The function of the falciform ‘hook-shape’ of the rodent sperm head and nucleus has been the subject of much debate. It has been suggested that the hook facilitates the formation of ‘sperm trains’ (Immler et al. 2007), in which a group of aggregated sperm are able to swim faster than an individual sperm. This is advantageous in species in which a female mates with multiple males in succession. However, directly associating sperm shape with functional advantages has been difficult, due to the wide ranging viscosities of the vaginal fluid in which they swim, the differing components to the seminal fluid, and differences in flagellar length and number (Simmons and Fitzpatrick 2012). Consequently, more research into sperm morphology and function is needed, especially into the processes that drive sperm head shape and by association, sperm nucleus shape.

### Associations of shape changes with fertility

Despite the variation in sperm shape, it is clear that there is an impact of shape abnormalities in fertility. Studies of the hydrodynamic efficiency of sperm from a range of different species have shown that sperm with morphological abnormalities are poorer swimmers, such as in humans (Katz et al. 1982; Gillies et al. 2009) and bulls (Ostermeier et al. 2001). Subsequent studies in cattle demonstrated that sperm motility varies between cattle breeds, and also varies depending on the temperature at which the sperm were developing (Rahman et al. 2011).

Morphological abnormalities are well-known contributors in human infertility; teratozoospermia, in which >85 % of sperm are morphologically abnormal, is frequently encountered in infertile men. The primary genetic correlates appear to be aneuploidies and DNA fragmentation (Braekeleer et al. 2015; Coutton et al. 2015). Mice with deletions on the long arm of their Y-chromosome exhibit abnormal morphologies, becoming more severe as the size of the deletion grows (Ward and Burgoyne 2006). Interestingly, sperm from males with this deletion also exhibit a sex-ratio skewing in favour of females, indicating that (in mice) there are different developmental effects of sex-linked genes on X-bearing and Y-bearing spermatids (Cocquet et al. 2012).

Clearly, there are important developmental pathways remaining to be elucidated in sperm development, especially those relating to the shaping of the sperm head, within and across taxa.

## Metakaryotic nuclei

Recently, there have been reports of a class of stem cells with large bell-shaped nuclei, present amongst human embryonic stem cells, and occasionally in adult adenocarcinomas (Gostjeva et al. 2006; Gostjeva et al. 2009). These ‘metakaryotic’ cells are suggested to divide syncytially, and to have an unusual chromosome pairing - with one chromosome arm condensed at the base of the bell, the second arm condensed at the mouth, and a short region including the centromere decondensed between them (Gruhl et al. 2010). However, the reported difficulties in preserving the nuclei without degradation make evaluating the importance or influence of such cells difficult at present.

## Spherical and ovoid nuclei

The majority of cell types encountered will have a spherical or ovoid nucleus. However, it is worth mentioning two examples of these as a functional contrast to particular morphologies covered above.

### T-lymphocytes

Lymphocytes are another migratory cell type that must pass through endothelial junctions. In T-lymphocytes, the nucleus is spherical and fairly rigid. Rather than flexibly deforming, it takes a forceful approach, squeezing the nucleus through the extracellular matrix using myosin-dependent contractions of the actin network behind the nucleus to force the rigid nucleus through narrow gaps (Lämmermann et al. 2008; Jacobelli et al. 2013).

### Pluripotent and embryonic stem cells

Stem cells are generally far more flexible than mature differentiated cells. The principle cause for this seems to lie in the composition of the nuclear lamina and lamin protein content; embryonic stem cells lack expression of lamins A/C, and epithelial cells with lamins A/C knockouts acquire a similar flexibility (Pajerowski et al. 2007). Rather than calling stem cells more flexible, then, perhaps a better phrasing is to say that during differentiation cells become more rigid. It seems that the hardening of nuclei is a response to mechanical stresses (Swift et al. 2013) and, thus, may be avoided in certain cell types that require flexibility for migration. In cells which are not motile, a sturdy lamina aids the structural integrity of the cell.

Supporting this, based on modelling of the spectrin-actin network in the erythrocyte plasma membrane, King and Lusk (2016) proposed that a stiffened nuclear lamina hinders chromatin remodelling, which we have seen accompanies cell type differentiation. Furthermore, embryonic stem cells have a nuclear stiffness that depends upon the level of compression experienced by the cell. This provides a mechanistic link between the cellular environment, and the regulation of the differentiation of the nucleus (Pagliara et al. 2014).

Embryonic stem cells also have a fascinating internal chromatin organisation. Bovine 8-cell embryos derived from IVF demonstrate clusters of predominantly peripheral chromatin (each cluster corresponding to a chromosome), with the central compartment of the nucleus remaining free of DNA (Popken et al. 2014). Cloned embryos derived from somatic cell nuclear transfer from fetal fibroblasts also show reorganisation of chromatin into a similar peripheral pattern, though generally with smaller central spaces than seen in IVF embryos. Popken et al. (2014) speculate that the central lacunae are used for storage of early factors required by the embryo, displacing the chromatin towards the nuclear periphery. As the embryonic genome activates (at the 10–16 cell stage), chromosomes reorganise according to gene-density, with the more gene-dense chromosomes migrating inward (Koehler et al. 2009).

## Invaginations of cytoplasm into nuclear channels

Alterations to nuclear morphology can be more subtle than described so far, yet have important functional roles. It has been known for many years now that interphase nuclei may be penetrated by invaginations of the nuclear membrane. The size and complexity of these invaginations vary between cell types, from short channels to long branched structures, to channels that pass from one side of the nucleus to the other entirely (Fricker et al. 1997).

Invaginations seem common across eukaryotes; they have been observed in the nuclei of plants, for example in onion and tobacco, and, as in the mammalian cells described above, the grooves and invaginations contain actin. They have been suggested to aid nucleocytoplasmic transport and signalling, and possibly calcium signalling (Collings et al. 2000).

As an example, skeletal muscle cells are typically spindle shaped, with an ovoid nucleus positioned at the periphery of the cell directly beneath the cell membrane (in contrast to the internal, fusiform nucleus of smooth muscle described above). Cells within the muscle fibre form a multinucleated syncytium, generated through the fusion of myoblasts during the development of the muscle. The nuclei have distinctive invaginations, channels that penetrate deep within its structure. These are filled with cytoskeletal components that are thought to facilitate trafficking of mRNAs to myofibrils (Abe et al. 2004).

Recent work using super-resolution microscopy has revealed the development of invaginations in bovine preimplantation embryos (Popken et al. 2015), which are hypothesised to assist transport into and out of the large nuclei, and may also be involved in the eventual reduction of nuclear volume and the shrinking of the nuclear envelope via formation of nuclear envelope vesicles.

There may be further roles for invaginations beyond transport; in mouse fibroblasts, deep invaginations are lost when the tissue is stretched, a change not thought to be attributable merely to the stretching and compression itself but instead suggesting a remodelling of the chromatin in response to the mechanical stress (Langevin et al. 2010).

However, it remains unclear how and when these invaginations develop in different lineages; invagination frequency may be linked to the activity of lamin B (Ellenberg et al. 1997; Popken et al. 2015), but how the channels form remains to be determined.

## Control of nuclear shape

Controlling the shape of the nucleus encompasses a variety of processes: the differentiation pathway of the cell, the mechanisms used to keep the shape stable and the mechanisms to alter the shape when needed.

For the differentiation of cell types, there is a major distinction between the somatic cell lineages, and those leading to gametes. The processes of meiosis and, in particular, chromatin condensation via the replacement of histones with protamines in sperm deserves separate consideration, and readers may consider recent reviews focussing on components in sperm shaping and chromatin organization (Xiao and Yang 2007; Rathke et al. 2014; O’Donnell and O’Bryan 2014).

**Fig. 3:**
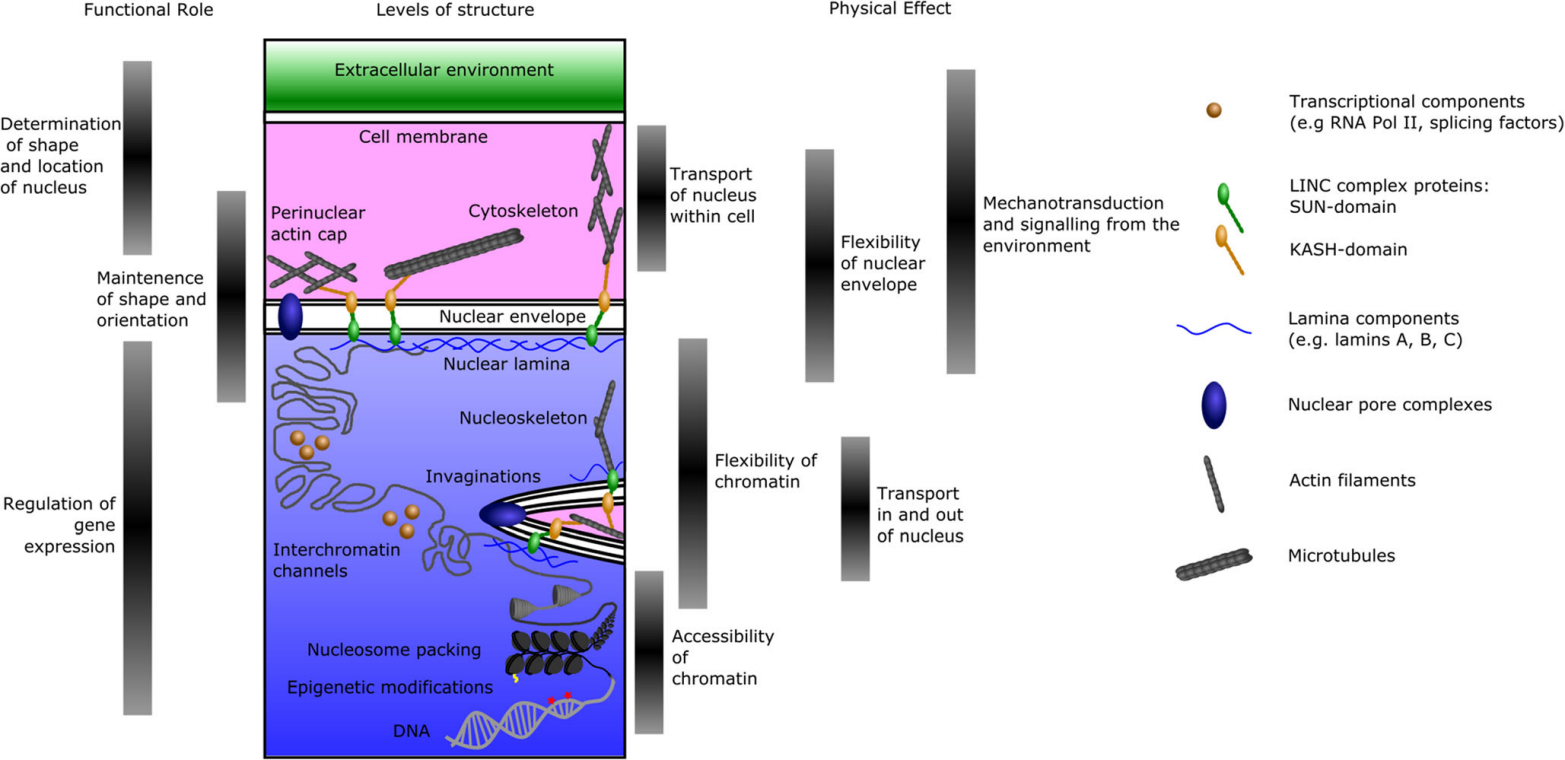
Layers of structure impacting nuclear shape, and their functional relevance. The levels of structure within a cell are schematically shown. Different ranges of structures have different effects upon the function of the cell, and are involved in different functional roles. The shape of the nucleus is determined by the cytoskeleton, the nuclear lamina, chromatin distribution and chromatin compaction. The nucleus can be repositioned and reoriented within the cell via actin- and microtubule-based transport, while mechanical stresses on the cell transmitted to the nucleus via the cytoskeleton can affect gene expression. Invaginations of the cytoplasm into the nucleus can provide additional transport for signalling molecules and RNAs

For the purposes of this review, we aim to provide only an overview of the components involved in shaping the nucleus, as given in Fig. 3. Readers interested in the details of the relevant components, and the available methods for measuring nucleus shape, size, stiffness and other parameters will be well served by the comprehensive reviews of nuclear structure and mechanics by (Webster et al. 2009; Lammerding 2011).

## Components involved in determining nuclear morphology

In brief, a large proportion of nuclear shape is determined by the interaction of the cell cytoskeleton with the nuclear envelope and the underlying nuclear lamina. The nuclear envelope consists of the double membrane surrounding the nucleus, with the nuclear pore complexes that permit transport across the membrane. The nuclear lamina lies beneath the inner membrane, and provides the majority of the structural support to the nuclear envelope (Burke and Stewart 2006).

Much of the mechanical linkage between the cytoplasm and the nucleus is mediated by the LINC complex (Linker of Nucleoskeleton and Cytoskeleton), composed of KASH-domain proteins and SUN-domain proteins. The KASH proteins cross from the cytoplasm through the outer nuclear membrane and into the perinuclear space. Here, they interact with SUN proteins, which pass from the perinuclear space through the inner nuclear membrane, to interact with the nuclear lamina, and via other molecular adapters, the nucleoskeleton (Tzur et al. 2006). External to the nucleus, the various KASH proteins can interact with cytoskeletetal components - for example, actin (nesprin-1G), intermediate filaments (nesprin-3α) and microtubules (nesprin-1, nesprin-2) (Chang et al. 2015).

Inside the nucleus, the SUN proteins bind lamin A, and more weakly lamins B1 and C (Crisp et al. 2006). The lamins are specialised intermediate filament proteins which form a mesh underlying the inner nuclear membrane; both the lamins and other nuclear-envelope-associated proteins are able to bind DNA and histones, thereby anchoring chromatin to the nuclear envelope (Czapiewski et al. 2016). Since the LINC complex connects the nucleoskeleton to the cytoskeleton of the cell, it provides a pathway to directly transmit mechanical forces which the cell is experiencing into stresses within the nucleus.

The nuclear envelope associated spectrin-repeat proteins (Nesprins) are KASH-domain proteins found within the LINC complex. They act as linkers within the nuclear envelope proteins, and to the cytoskeleton or the nucleoskeleton (Rajgor and Shanahan 2013). The role of spectrins outside the nucleus has been well studied; they help to provide the flexibility and elasticity that smooth muscle nuclei and other organelles require during contraction (Wang and Volk 2015; King and Lusk 2016). They also appear to have a role in the development of nuclear shape in sperm; in falciform sperm, such as from rats, spectrin has been found to be associated with the apical hook (Dvořáková et al. 2005), suggesting a contribution to the development of the sperm shape, as well as a functional contribution to capacitation and the acrosome reaction (Bastián et al. 2010).

## Nuclear shape from within

In addition to the stiffness or flexibility from the nuclear lamina and envelope, the deformability of the nucleus is affected by the flexibility of the chromatin it contains. Recent work by Schreiner et al. (2015) in the fission yeast *Schizosaccharomyces pombe* showed the effect of chromatin flow on the rigidity of the nucleus. *S.pombe* lacks lamins, and thus has a flexible nucleus which can be studied outside the influence of the lamin proteins. Schreiner et al. (2015) found that the degree of tethering of chromatin by LINC complex proteins to the inner nuclear membrane affected the stiffness of the nucleus against microtubule-induced deformations.

BRG1 (Brahma-related gene 1) is an ATPase subunit of the SWI/SNF chromatin remodelling enzyme complex. Depletion of BRG1 in mammary epithelial cells was observed to increase lobation of the nucleus, independently of cytoskeletally related effects. BRG1 is able to affect gene expression as well as chromatin structure, and consequently, it appears that the overall shape of the nucleus can be determined internally, as well as from without (Imbalzano et al. 2013).

The protein component of the nuclear envelope is unsurprisingly the focus of many studies. However, the lipid component is also relevant. Polychronidou and Großhans (2011) reviewed the role of farnesylation of proteins on nuclear shape, and point out that the rate of lipid insertion into the nuclear membrane can affect nuclear envelope size, and that farnesylation of lamins and their *Drosophila* homologues results in nuclear shape abnormalities. This may be mediated by insertion of the proteins into the nuclear lipid membrane and suggests that in addition to the interactions of the nuclear envelope with cytoskeletal proteins, deformation of the nuclear envelope is also driven by an interaction of the phospholipid bilayer with farnesylated membrane proteins. Nuclear shape abnormalities may therefore be linked to defects in lipid biosynthesis. Supporting the role of lipids, recall the neutrophil hypersegmentation in Boucher-Neuhäuser syndrome (Koh et al. 2015); the responsible gene is *PNPLA6*, which encodes an enzyme responsible for deesterification of membrane phosphatidylcholine (Synofzik et al. 2014). The breakdown of a major membrane phospholipid is clearly important, though the mechanism from *PNPLA6* defects to hypersegmentation has not yet been elucidated.

The rate at which lipids are inserted into the nuclear membrane is also affected by the determination of whether new fatty acids generated at the endoplasmic reticulum are destined to become phospholipids (for membranes) or triacylglycerols (for energy storage). Lipins are involved in the production of triacylglycerols, and thus defects in the lipins can lead to overproduction of membrane lipids and expansion of the nuclear membrane (Santos-Rosa et al. 2005). The resulting nuclear morphological defects have been observed in both yeasts (Barbosa et al. 2015) and *Drosophila melanogaster* (Ugrankar et al. 2011), manifesting as an increased nuclear size, involutions and projections from the nuclear envelope.

### Chromatin organisation within the nucleus

It has long been appreciated that chromosomes occupy distinct territories within interphase nuclei, often with preferred nuclear addresses for loci, as recently reviewed (Cremer and Cremer 2010; Bickmore and van Steensel 2013). The positioning of chromatin can have a direct physical impact on cellular function. Chromatin compaction into heterochromatin and euchromatin can itself provide structural support for the nucleus. The organisation of chromatin within the nucleus—especially the relative locations of homologous chromosomes—can affect rates of double-strand break repair, and processes such as non-allelic recombination (Agmon et al. 2013). These are drivers for both evolution (in the germline) and disease (in somatic cells).

In a more dramatic example of structure linking to function, mammalian retinal neurons show a gross reorganisation of chromatin in nocturnal mammals compared to diurnal mammals. In nocturnal mammals, the shapes of the nuclei in the rod cells are elongated ellipses (Błaszczak et al. 2014), and the internal chromatin is dramatically altered. The standard arrangement of peripheral heterochromatin and internal euchromatin is inverted (Solovei et al. 2009). This change to the distribution of chromatin density through the nucleus appears to provide an advantage in the focussing of light within the retina, and has independently evolved multiple times in mammalian evolution.

## Nuclear shape from without

While some changes to nuclear shape are directed from within the nucleus itself, other changes are imposed onto the nucleus by external forces. Cytoskeletal tension transmitted to the nucleus can directly affect the phosphorylation of lamins; this in turn affects the rate of lamin turnover and activity, leading to a softening of the nuclear envelope as tension on the nucleus decreases, and a more rounded morphology (Buxboim et al. 2014). Consequently, the local tissue environment can affect the morphology that the nucleus will adopt. Observations of mesenchymal stem cells grown on various extracellular matrices demonstrated that the stiffness of the underlying matrix affects tension upon the nucleus and influences both the nuclear shape and the differentiation pathway of the cell (Swift et al. 2013). Gene expression changes following mechanical stresses seem to be required to trigger particular developmental pathways, as in the transition from mammary epithelium to mesenchyme (Nelson et al. 2008).

We have described the requirement for flexible nuclei in granulocytes and monocytes, to facilitate migration of cells from cells into tissues. The process of migration to a new environment can impose a purely mechanical change of nuclear shape on any cell. In cells with active forces impacting on the nucleus, the nuclear envelope proteins are required to resist these forces, and maintain the appropriate shape (Webster et al. 2009). Deformations due to motion can be large; Martini and Valdeolmillos (2010) show the nuclei of mouse cortical interneurons being moved by actomyosin contractions immediately behind the nucleus. Nuclei can also be moved in a saltatory manner along microtubules by dynein (Tsai et al. 2007), resulting in significant deformation of shape during motion. McGregor et al. (2016) point out that mechanical stresses on nuclei can physically damage the nuclear envelope and DNA.

Damage which does occur to the nuclear envelope can permit exchange of material between the nucleoplasm and cytoplasm, as well as leading to chromatin spilling from the nucleus and subsequent DNA damage. As the nucleus is deformed, damage to the nuclear envelope must be repaired, and the Endosomal Sorting Complexes Required for Transport III (ESCRT III) machinery appears to be important for this process (Denais et al. 2016). The ESCRT III complex is also involved in reformation of the nuclear envelope following mitosis (Olmos et al. 2015). Ruptures of the nuclear envelope in migrating HeLa cells have recently been observed (Raab et al. 2016), and this work has demonstrated that repair of the envelope is dependent on the ESCRT III complex. Consequently, it is likely that this mechanism will turn out to have further implications in pathologies among migratory cells.

We noted above that the chromatin compaction helps influence the stiffness of the cell. Measurements of isolated nuclei suggest that the cell cytoskeleton can oppose chromatin compaction, holding the nucleus open (Mazumder and Shivashankar 2007), showing a balance between the internal and external forces within the nucleus. The impacts of this tension are not well established, but could conceivably affect access of genes to transcription factories and thereby influence gene expression.

## Nuclear position within the cell

Viewing the nucleus as an organelle within the cell, how is the nuclear orientation established and maintained? What is the relationship between the orientation or polarity of the cell and the orientation of the nucleus within the cell?

Regarding the positioning of the nucleus, the answer seems clear. Motor protein complexes are involved in rotating and positioning nuclei within cells. Gerashchenko et al. (2009) showed that active nuclear rotation depends on dynein and microtubules, while vimentin intermediate filament proteins act to stabilise nuclear orientation and connect the nucleus to the cytoskeleton.

Regarding the importance of the nucleus in cell polarity, evidence is less clear. Cell polarity seems to depend more on the position of the centrosome than of the nucleus. In lymphocytes, for example, the centrosome is decoupled from the nucleus before the microtubule network of the cell is reorganised to establish cell polarity (Lui-Roberts et al. 2012; Obino et al. 2016). We have previously seen that migrating neutrophils can have a nuclear orientation provided through the inactive X chromosome, but the means by which this translates into cell orientation appears to still be via the centrosome (Yoo et al. 2012). Whether the nuclear orientation is involved in establishing cell polarity remains unknown.

Working with *Drosophila melanogaster* embryos, Ramdas and Shivashankar (2015) found knockdowns of actin-associated linker proteins resulted in disruption of the nuclear position within the cell. In contrast, knockdown of microtubule-associated proteins had no such effect. Alterations to nuclear morphology were, as in other studies, accompanied by gene expression changes.

## When is nuclear shape determined during cell division?

Some alterations to nuclear shapes occur in a terminally differentiated cell. These are the changes seen for example in macrophages. Other morphologies are established as the cell exits mitosis from a precursor with a different nuclear shape.

After the nuclear envelope reforms, chromosomes must adopt their preferred organisations and chromatin densities within the new nucleus. It is still poorly understood how this is mediated, and over what timescales it occurs. Webster et al. (2009) point out possible scenarios for this; in the first, the chromatin is non-randomly organised at telophase, and this determines the chromatin-nuclear envelope contacts that will be established. In the second scenario, chromatin organisation is random at the point of nuclear envelope formation, and then organises into a preferred configuration. Reality may of course also be a mix of these scenarios, dependant on cell type and the locus of interest.

Studies of chromatin dynamics through mitosis using chromatin conformation capture based approaches such as 5C and Hi-C have suggest that metaphase chromosomes from different cell types have similar organisations (Naumova et al. 2013). The implication is that cell-type specific organisations are adopted post-mitotically, driven by the transcriptional and epigenetic environment of the cell.

## Nuclear shape can affect gene expression

In many cell types, heterochromatin is attached to the nuclear lamina at the periphery of the nucleus, and the active euchromatin is towards the interior. It has been observed that individual loci and gene clusters can move towards the interior of the nucleus upon transcriptional up-regulation (Stadler et al. 2004), though locus repositioning does not always correlate with gene activity (Meaburn and Misteli 2008). Furthermore, tethering a normally interior chromosome territory to the nuclear periphery can result in a downregulation of some (though by no means all) of the genes it contains (Finlan et al. 2008). Consequently, the shape of the nucleus will affect the amount of chromatin brought in proximity to the nuclear lamina and may thereby further affect gene expression.

A more direct link between nuclear shape and gene expression can be seen mediated by mechanotransduction. In fibroblasts, nuclear shape is controlled by an actin cap across the top of the nucleus, which enables the cell to regulate the shape of the nucleus according to the underlying surface to which the cell adheres (Khatau et al. 2009). These actin-related shape changes begin a broader alteration to the cell’s transcriptional profile in response to cell shape change. One mechanism underlying such gene expression changes is the active transport of transcription cofactors such as transcriptional repressor histone deacetylase 3 (HDAC3) and myocardin-related transcription factor (MTRF-A) in an actomyosin dependent manner (Jain et al. 2013).

Studies across many different cell types have found that nuclear deformations are not just able to induce expression changes, but may be *required* to establish proper transcriptional profiles. We have mentioned previously the impact of the cell substrate stiffness on differentiation from epithelium to mesenchyme. In further examples, actin-dependent deformation is required for CD69 expression in naive T-lymphocytes (Gupta et al. 2012). Collagen I synthesis has been linked with a particular range of nuclear deformations, and expression of the bone differentiation marker osteocalcin was increased in cells with a constrained nucleus over cells with no such constraints (Thomas et al. 2002).

Studies measuring nuclear shape in cells seeded at different densities show rounder nuclei in cells at high densities than at lower densities, with corresponding increases in the expression of genes involved in chromatin condensation (McBride and Knothe Tate 2008), further demonstrating the link from the population-level environment of the cell to the shape and activity of the nucleus.

## Nuclear shape and structure in pathology

### Cancers

When considering diseases frequently associated with morphological changes in the nucleus, the cancers must be at the top of the list. Histopathologists have long known that one of the diagnostic features of neoplasia is pleomorphy, an increased variation in nuclear size and shape. Pleomorphy becomes more severe as tissues progress towards carcinomas. Clearly, the normal processes controlling nuclear morphology are disrupted. Accordingly, when Bussolati et al. (2008) studied pleomorphies in breast cancer samples via lamin B and emerin staining, they observed intranuclear deposits forming a scaffold, which they attributed to the formation of intranuclear tubules, indicating defects nuclear envelope structure, and potentially transport between the nucleus and cytoplasm. More recently, Funkhouser et al. (2013) have shown that alterations to the mesh size of the lamin A component of the nuclear lamina can also mechanistically result in blebbing of the nucleus. We note here again that the functional impact of the lamins for structure is clear from the earlier consideration of neutrophil defects, and in the flexible stem cells lacking lamins A/C; the disease impact of lamin defects, the laminopathies, are also well documented (Burke and Stewart 2006; Lammerding 2011).

Both nuclear morphology and size are used clinically for the diagnosis of various cancers; a histopathologist will identify dysplastic tissue and subsequent carcinomas by the proportion of irregularly shaped cells and nuclei in an H&E-stained tissue section as well as by the frequency of cell division. Specific carcinomas have their own distinguishing features—a recent example demonstrates urothelial carcinomas may be distinguished from other urothelium by their particularly large nuclei (Poropatich et al. 2016). For a clinician, this may be a valuable tool for prognosis, as well as for diagnosis; morphological features of nuclei such as their symmetry and size can indicate how a patient may respond to chemotherapy. Such studies have been ongoing for a long time—see, for example, an assessment of the utility of nuclear shape for prognosis in prostate cancers (Diamond et al. 1982), through to a recent study identifying squamous cell carcinomas by measures such as nuclear area, compactness, symmetry and sharpness of the edge (Ogura et al. 2015).

Morphological changes in cancers affect both the shape of the nucleus, and the composition and behaviour of the nuclear envelope. A recent review of the nuclear envelope (Bell and Lammerding 2016) points out that lamin content of the nuclear envelope is altered in some cancers, most notably a reduction in lamin A/C levels. As described for migratory cells earlier, this can increase the flexibility of the nuclear envelope, and facilitate penetration of the cells through the extracellular matrix. It should also be noted that variation in the composition of the nuclear envelope can affect signalling pathways and may contribute to the transcriptional differences in cancers.

Cellular senescence is a terminal arrest of the cell cycle, caused by factors such as telomere shortening, oxidative stress, DNA double-strand breaks and oncogene activation (Dolivo et al. 2016). In fibroblasts, senescence is marked by a profound rearrangement of chromatin, with formation of prominent heterochromatin foci throughout the nucleus (Narita et al. 2003). These foci result from an inversion of chromosome territory structure, with densely packed constitutive heterochromatin surrounded by facultative heterochromatin and then euchromatin; this organisation may stabilise the expression levels of particular genes, at the cost of transcriptional flexibility (Chandra et al. 2012).

Attempts are being made to use such modification of the epigenome clinically. Chaetocin is a promising candidate therapeutic agent for treating of tumuors (e.g. Lai et al. 2015; Jung et al. 2016). It is an inhibitor of a histone methyltransferase (mediating trimethylation of histone H3 lysine 9 (H3K9m3), a marker of constitutive heterochromatin and repressed chromatin). Treatment of fibroblasts with chaetocin results in chromatin reorganisations: nuclei form clusters of condensed chromatin, similar to the foci seen in senescent cells (Illner et al. 2010). In these treated cells, gene-dense chromatin repositioned to the periphery of the chromosome territory, though expression data for relevant genes has yet to be obtained.

### Hutchinson–Gilford progeria syndrome

The laminopathies have been mentioned briefly already. One in particular bears mentioning here, Hutchinson–Gilford progeria syndrome. This results from defects in lamin A, with consequences for the structural stability of the nucleus, and its gene expression (Vidak and Foisner 2016). Nuclei frequently have a thickened lamina (Worman and Courvalin 2005), and are less resistant to mechanical stress (Zhang et al. 2011). The symptoms appear much like an accelerated form of normal aging, with attrition of telomeres and premature senescence of cells (Burtner and Kennedy 2010).

Given the description of senescence in the section above, one might expect this progeria syndrome to show a similar phenotype. However, while cells from patients do have an alteration to their epigenome including reduced H3K9me3 (Shumaker et al. 2006), they lack condensed heterochromatic foci. Further investigation has shown that both processes do share features: a decondensation of particular AT-rich lamina-associated chromatin domains, and an inversion of the chromatin patterns seen in embryonic stem cells. However, the progeric cells do not progress to form heterochromatin clusters (Chandra et al. 2015). This indicates two distinct processes are involved: firstly, the disruption of chromatin interactions with the nuclear lamina; secondly, the clustering of these heterochromatic regions specifically in senescent cells. A full understanding of how this additional change in nuclear architecture affects function in senescent versus progeric cells remains to be determined.

### Viral infection of cells

Nuclear morphology and size can alter greatly upon infection by viruses. For example, infection of HeLa cell lines by the herpes simplex virus 1 (HSV-1) results in the virus occupying the channels and spaces between chromosomes (Monier et al. 2000). These spaces are expanded as the virus replicates, until the mature virions are released from the nucleus, with the nuclear volume doubling during this process. Evidence from many viral families now suggests that interactions with the nuclear actin, the nuclear lamina and other nucleoskeletal components are necessary for viral capsid formation and exit from the nucleus (Cibulka et al. 2012). Furthermore, the expansion of the nucleus upon infection requires disruption of the nuclear lamina, mediated by viral proteins (Simpson-Holley et al. 2005).

## Conclusions and perspectives

Many descriptions of nuclear shapes have come from two-dimensional imaging and analysis. Of course, the cells are three dimensional objects and are deformed when dropped onto a slide. How closely do the structures, shapes and behaviours we have seen in two dimensions resemble the true organisation of the living cell? Live cell imaging and three-dimensional microscopy are allowing us to make these comparisons for a number of cell types and show that 3D matters: fibroblasts with lamin defects can exhibit normal 2D motility, but in a 3D matrix their movement is impaired (Versaevel et al. 2013). The fractal organisation of chromatin in 2D and 3D affects transcription factor diffusion and binding (Woringer et al. 2014), and recent insights from prokaryotic systems into the complex regulatory logic, deriving from transcription factor access to chromatin (Ezer et al. 2014), show that a comprehensive understanding of transcriptional regulation in eukaryotes driven by nuclear architecture requires a three-dimensional viewpoint.

For all the information, we gain on chromatin structure looking at carefully fixed cells in two or three dimensions, we still do not get a good idea of how cells are actually behaving. Imaging of the nuclear dynamic of live cells has already revealed some fascinating insights into the stretching and squeezing of a nucleus in migrating cells (e.g. Versaevel et al. 2012; Yoo et al. 2012). These experiments will become more powerful as culturing systems are developed, such as 3D scaffolds that can better mimic the in-vivo environment (Li and Kilian 2015). The production of cell lines with fluorescent DNA labels incorporated has already been demonstrated for cell cycle analysis (Sakaue-Sawano et al. 2008), and the current favourite gene editing technology, CRISPR/Cas9, provides a means to target labels to particular genomic loci and track them in living cells (Chen et al. 2013).

Many of the imaging studies performed to date, in either 2D or 3D, require careful setup and manual imaging of a small number of cells. One of the challenges for the future is to extend these analyses to greater numbers of cells to better appreciate the variation within cell populations. Automated image analysis methods are available and have been successfully used for studies of nuclear morphology (e.g. Ballarò et al. 2008), but these are frequently 2D.

Assessing the 3D chromatin structure will involve a combination of detailed imaging (e.g. Schreiner et al. 2015) with high-throughput techniques such as chromosome conformation capture and its variants, in which chromatin structure can be reconstructed by sequencing and mapping physically adjacent regions of DNA. Although chromosome conformation capture has the limitation of operating at the scale of millions of cells, and averages out subtle differences between individual cells, it has demonstrated clear and reproducible differences between cell types (Pueschel et al. 2016). Extending chromatin capture based approaches to single cell genomic analysis is unlikely to work robustly due to the low genome coverage per cell, but single cell analysis is developing fast for both transcriptional and epigenomic studies (Trapnell 2015).

In conclusion, the variety of morphologies that nuclei can adopt has clear functional impacts and important roles in controlling the activity of the cell. The coming years will provide an unprecedented resolution with which to study genome organisation and a far better understanding of how nuclear morphology regulates and is regulated by the activity and environment of the cell.
